# hsa-miR-29c-3p regulates biological function of colorectal cancer by targeting SPARC

**DOI:** 10.18632/oncotarget.22356

**Published:** 2017-11-10

**Authors:** Shitong Zhang, Jianjun Jin, Xiaoxiao Tian, Lijuan Wu

**Affiliations:** ^1^ First Affiliated Hospital of Henan University of Science and Technology, Luoyang City 471003, Henan Province, China

**Keywords:** colorectal cancer, SPARC, hsa-miR-29c-3p, proliferation, migration

## Abstract

Colorectal cancer (CRC) is the most common type of behavioral cancers, miRNAs play a critical role in cancer development and progression. In the present study, we downloaded the original data from Gene Expression Omnibus (GEO) and conduct data analysis. has-mir-29c-3p mimic, inhibitor, negative control or si-SPARC (secreted protein acidic, rich in cysteine) were transfected into HCT116 cells, respectively. Quantitative real time PCR (qRT-PCR) was used to measure has-mir-29c-3p and SPARC mRNA expressions, western blot was used to detect ACAA1 (acetyl-CoA acyltransferase 1), ACOX1 (acyl-CoA oxidase 1), COL1A1(collagen, type I, alpha-1), COL1A2 (collagen, type I, alpha-2), COL4A1 (collagen, type IV, alpha-1), COL5A2 (collagen, type V, alpha-2), COL12A1 (collagen, type XII, alpha-1), CPT2 (carnitine palmitoyltransferase 2), ETHE1 (persulfide dioxygenase), HMGCS2 (3-hydroxy-3-methylglutaryl-CoA synthase 2), SPARC, SQRDL (sulfide quinone oxidoreductase), and TST (thiosulfate sulfurtransferase) protein expression. CCK-8 and wound healing assay were employed to verify cell proliferation and migration. The luciferase reporter assay data made sure the target correlation of has-mir-29c-3p and SPARC. Firstly, we found that the expression of has-mir-29c-3p was lower in CRC tissues than in their paired corresponding non-cancerous tissues and there was significant inversed correlation between has-mir-29c-3p and SPARC. Overexpression of has-mir-29c-3p reduced cell proliferation and migration. SPARC was identified as a direct target of has-mir-29c-3p, whose silencing reduced cell proliferation and migration. These data showed that has-mir-29c-3p regulates CRC cell functions through regulating SPARC expression. Taken together, has-mir-29c-3p may function as an oncogenic miRNA targeting SPARC, targeted modulation of has-mir-29c-3p expression may became a potential strategy for the treatment.

## INTRODUCTION

Colorectal cancer (CRC) is the third most common cancer, and it is the forth leading cause of cancer-related deaths in the world [1, 2]. Between one million and two million new cases are diagnosed each year, and more than 700 000 people die each year [3, 4]. According to gender, it is the second most common cancer in women and the third in men. The global incidence rate is as high as 10 times, with almost 55% of cases occurring in more developed areas. In most cases, CRC was detected in western countries (55%), but this trend is changing due to the rapid development of some countries in the past few years. Local recurrence and distant metastasis are the main causes leading to the death of CRC [5]. Therefore, understanding of the factors involved in CRC metastasis is necessary for identifying new biomarkers and for developing new anti cancer strategies.

Recently, microarray technology has been developing rapidly and has been widely used to reveal the general genetic alteration in progression of diseases, which enables the identification of targets for diagnosis, therapeutic, and prognosis of tumors. miRNAs are a group of non coding RNAs, found in Caenorhabditis elegans in 1993, and are now documented in many other organisms. They act in target messenger RNAs, affecting protein translation [6, 7]. They are short and highly conserved, down-regulation of gene expression through its association with the 3’-untranslation regions (3’ UTRs) at the post transcriptional level and plays important roles in physiological and pathological processes [8, 9], particularly in cancer development and progression [10, 11]. miRNAs may act as a tumor suppressor gene or oncogene, and may regulate tumor invasion and metastasis such as the EMT. [12, 13], which may depend on whether it is deleted or overexpressed [14-16]. At present, the role of miRNA in the key steps of CRC metastasis is still unclear. Recently, it has been found that miR-29c is a tumor suppressor miRNA and the down regulation of miR-29c was found in several types of cancer, including breast, lung, stomach, liver cancer [17, 18]. However, it has been implicated that miR-29c may be associated with recurrence and metastasis of colon cancer [19], more details about the mechanisms and pathways involved in miR-29c function need to be validated.

SPARC, also known as bone adhesion protein and basal membrane-40 protein, is a member of the extracellular matrix protein family [20]. Thus, this protein governs the basic functions of cells, such as cell adhesion, proliferation and differentiation [21]. The expression of SPARC was first identified in bone and endothelial cells and was also highly expressed in embryonic tissues, and played roles in the development and differentiation of chondrocytes and megakaryocytes [22, 23]. SPARC has a wide range of biological effects [24]. Rapid proteolysis of SPARC via various proteases, including matrix metalloproteinases (MMPs), plasmin and trypsin, in order to prevent the rapid accumulation of proteins in the extracellular environment [25]. SPARC is also expressed in many advanced cancers and associated with tumor migration and invasion. Recently, the up-regulated expression of SPARC was associated with gastric cancer, esophageal cancer and CRC [23, 26, 27], and this high levels of SPARC have been shown to be associated with poor prognosis in gastric cancer [27]. All of these indicated that SPARC played an important role in cancers.

In the present study, we downloaded the original data from Gene Expression Omnibus (GEO, http://www.ncbi.nlm.nih.gov/geo), and conduct data analysis. Explored the relationship between SPARC, miRNA in CRC. We try to identify a novel has-mir-29c-3p target gene, SPARC, which is involved in invasion and migration of colon cancer cells and further supports the role of mir-29c-3p in the inhibition of cancer in human cancers.

## RESULTS

### Identification of DEGs

**Figure 1:**
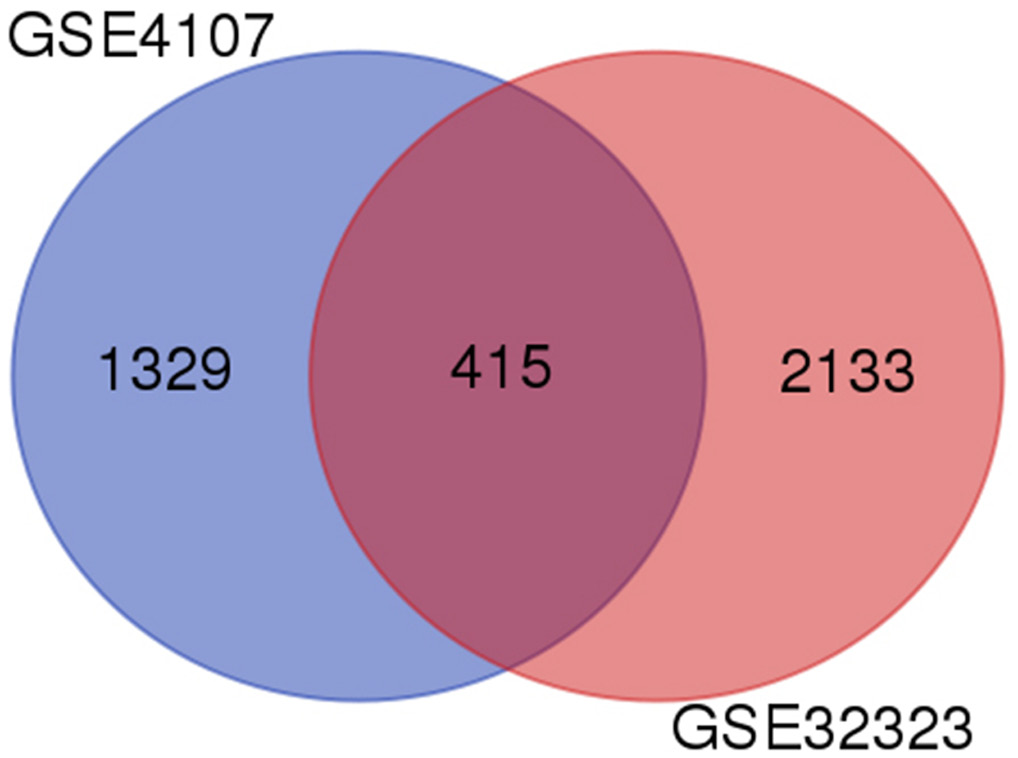
Identification of differentially expressed genes in 554 mRNA expression profiling datasets GSE4107 and GSE32323

A total of 1744 and 2548 DEGs were identified from GSE4107 and GSE32323 datasets, respectively. 415 genes were screened out in all two datasets (Figure 1). These genes were common different genes. Among them, 183 genes presented identical expression trends in all two datasets, consisting of 61 up-regulated genes and 122 down-regulated genes in CRC tissues compared to healthy colorectal tissues.

### Functional and pathway enrichment analysis

We uploaded all DEGs to the online software DAVID to identify overrepresented GO categories and KEGG pathways. The up-regulated genes were mainly involved in biological processes (BP) associated collagen fibril organization, cellular response to amino acid stimulus, basement membrane organization, skin morphogenesis and protein heterotrimerization (Table 1). For cell component (CC), the up-regulated DEGs were enriched in extracellular space, proteinaceous extracellular matrix, collagen type I trimer, collagen trimer and extracellular exosome, and the down-regulated DEGs were enriched in extracellular exosome, integral component of membrane, mitochondrial inner membrane, brush border and cytosol (Table 1). In addition, GO molecular function (MF) analysis also displayed that the up-regulated DEGs were significantly enriched in extracellular matrix structural constituent and extracellular matrix binding, and down-regulated DEGs enriched in oxidoreductase activity, flavin adenine dinucleotide binding, electron carrier activity, actin filament binding and acyl-CoA dehydrogenase activity (Table 1). Moreover, four KEGG pathways were over-represented in protein digestion and absorption, ECM-receptor interaction, amoebiasis, focal adhesion and PI3K-Akt signaling pathway, and down-regulated DEGs enriched in sulfur metabolism, metabolic pathways, fatty acid degradation, starch and sucrose metabolism and drug metabolism - cytochrome P450 (Table 2).

**Table 1:** Gene ontology analysis of differentially expressed genes associated with CRC

Expression	Category	Term	Description	Gene count	P value
Up-regulated	GOTERM_BP_DIRECT	GO:0030199	collagen fibril organization	5	3.77E-06
	GOTERM_BP_DIRECT	GO:0071230	cellular response to amino acid stimulus	4	4.37E-04
	GOTERM_BP_DIRECT	GO:0071711	basement membrane organization	2	0.02585
	GOTERM_BP_DIRECT	GO:0043589	skin morphogenesis	2	0.02585
	GOTERM_BP_DIRECT	GO:0070208	protein heterotrimerization	2	0.029489
	GOTERM_CC_DIRECT	GO:0005615	extracellular space	12	2.06E-05
	GOTERM_CC_DIRECT	GO:0005578	proteinaceous extracellular matrix	5	0.001402
	GOTERM_CC_DIRECT	GO:0005584	collagen type I trimer	2	0.006026
	GOTERM_CC_DIRECT	GO:0005581	collagen trimer	3	0.00709
	GOTERM_CC_DIRECT	GO:0070062	extracellular exosome	13	0.018026
	GOTERM_MF_DIRECT	GO:0005201	extracellular matrix structural constituent	4	2.14E-04
	GOTERM_MF_DIRECT	GO:0050840	extracellular matrix binding	2	0.070803
Down-regulated	GOTERM_BP_DIRECT	GO:0006805	xenobiotic metabolic process	6	1.22E-04
	GOTERM_BP_DIRECT	GO:0033539	fatty acid beta-oxidation using acyl-CoA dehydrogenase	4	1.76E-04
	GOTERM_BP_DIRECT	GO:0055114	oxidation-reduction process	12	0.001093
	GOTERM_BP_DIRECT	GO:0006654	phosphatidic acid biosynthetic process	4	0.001308
	GOTERM_BP_DIRECT	GO:0006635	fatty acid beta-oxidation	4	0.002542
	GOTERM_CC_DIRECT	GO:0070062	extracellular exosome	35	4.89E-05
	GOTERM_CC_DIRECT	GO:0016021	integral component of membrane	50	3.38E-04
	GOTERM_CC_DIRECT	GO:0005743	mitochondrial inner membrane	10	0.0016
	GOTERM_CC_DIRECT	GO:0005903	brush border	4	0.005996
	GOTERM_CC_DIRECT	GO:0005829	cytosol	32	0.008631
	GOTERM_MF_DIRECT	GO:0016491	oxidoreductase activity	9	3.07E-05
	GOTERM_MF_DIRECT	GO:0050660	flavin adenine dinucleotide binding	6	4.39E-05
	GOTERM_MF_DIRECT	GO:0009055	electron carrier activity	6	2.23E-04
	GOTERM_MF_DIRECT	GO:0051015	actin filament binding	6	0.001286
	GOTERM_MF_DIRECT	GO:0003995	acyl-CoA dehydrogenase activity	3	0.004184

If there were more than five terms enriched in this category, top five terms were selected according to P value. Count: the number of enriched genes in each term.

**Table 2:** KEGG pathway analysis of differentially expressed genes associated with NSCLC

Expression	Pathway ID	Description	Gene count	P value	Genes
Up-regulated	04974	Protein digestion and absorption	5	5.87E-05	COL4A1, COL1A2, COL12A1, COL1A1, COL5A2
	04512	ECM-receptor interaction	5	6.74E-05	COL4A1, COL1A2, COL1A1, COL5A2, SPP1
	05146	Amoebiasis	5	1.46E-04	COL4A1, COL1A2, CXCL8, COL1A1, COL5A2
	04510	Focal adhesion	6	1.56E-04	COL4A1, COL1A2, COL1A1, COL5A2, PARVB, SPP1
	04151	PI3K-Akt signaling pathway	6	0.001474	COL4A1, COL1A2, COL1A1, GNG4, COL5A2, SPP1
Down-regulated	00920	Sulfur metabolism	6	1.53E-08	TST, SQRDL, ETHE1, CYCS, BPNT1, PAPSS2
	01100	Metabolic pathways	27	1.96E-05	ACOX1, PLD1, HSD17B2, ACADS, ENPP3, MAOA, NAT1, CYCS, NAT2, ADH1C, ADH6, GPAT3, CMBL, TST, GBA3, MTM1, PLCE1, HMGCS2, AKR1B10, PGM1, ETNK1, UGT2A3, AHCYL2, PAPSS2, BPNT1, UGP2, ACAA1
	00071	Fatty acid degradation	6	5.16E-05	ACOX1, CPT2, ACADS, ADH1C, ADH6, ACAA1
	00500	Starch and sucrose metabolism	4	0.003544	GBA3, ENPP3, PGM1, UGP2
	00982	Drug metabolism - cytochrome P450	5	0.0037	FMO5, MAOA, ADH1C, ADH6, UGT2A3

If there were more than five terms enriched in this category, top five terms were selected according to P value. Count: the number of enriched genes in each term.

### PPI network construction and modules selection

**Figure 2:**
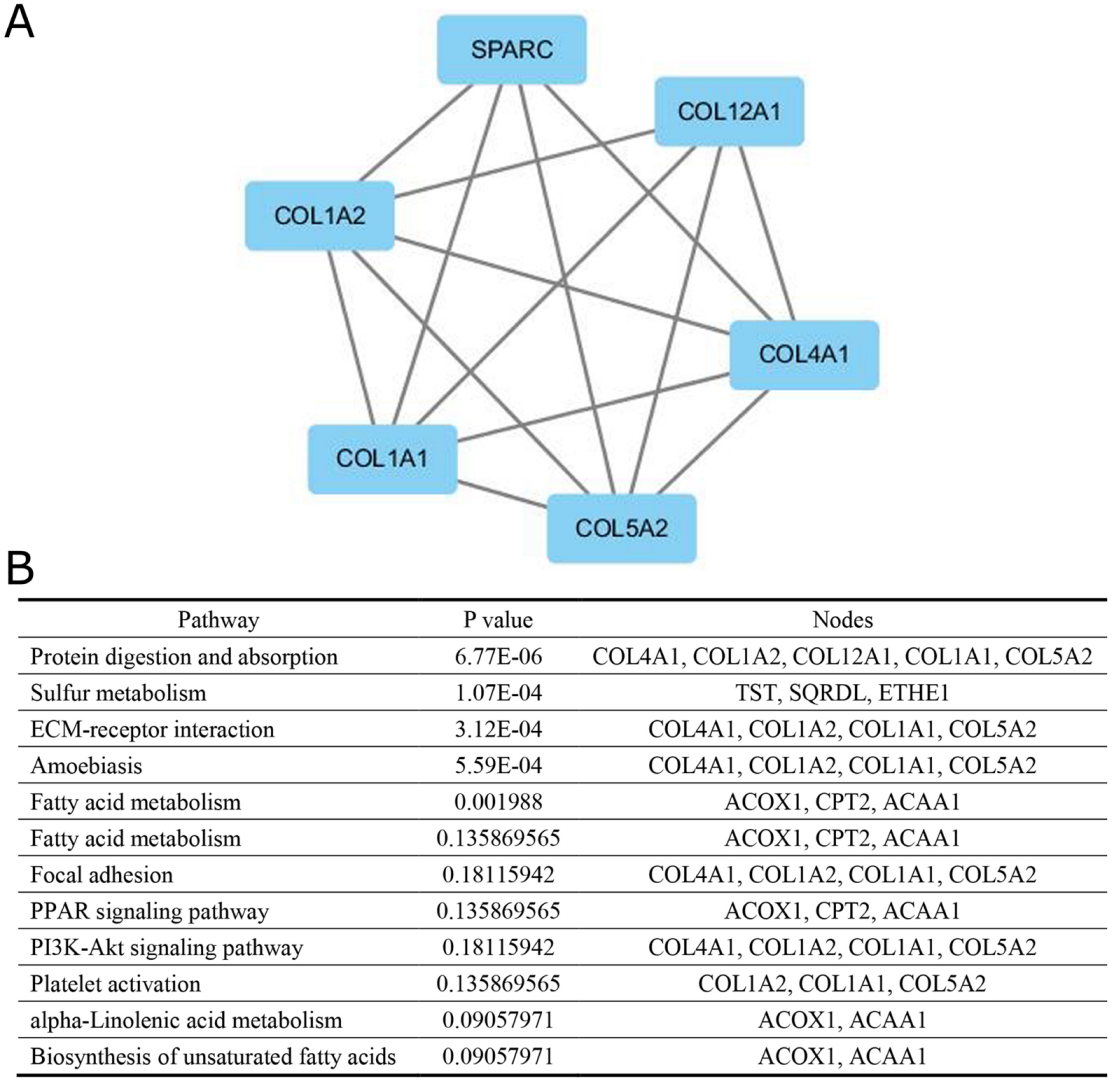
The protein–protein interaction network **(A)** a module, **(B)** the enriched pathways of this module.

A significant module was obtained from PPI network of DEGs using MCODE, including 6 up-regulated genes (Figure 2A) and 7 down-regulated genes. Functional and KEGG pathway enrichment analysis revealed that genes in this module were mainly associated with protein digestion and absorption, sulfur metabolism, ECM-receptor interaction, amoebiasis, fatty acid degradation, fatty acid metabolism, focal adhesion, PPAR signaling pathway, PI3K-Akt signaling pathway, platelet activation, alpha-Linolenic acid metabolism and biosynthesis of unsaturated fatty acids (Figure 2B).

### DEG – miRNA pairs

A total of 13 differentially expressed mRNAs were screened, consisting of 6 up-regulated and 7 down-regulated mRNAs. As shown in Table 3, hsa-miR-29c-3p was common in 5 up-regulated genes, while there was no identical miRNA in the down-regulated genes.

**Table 3:** Differentially expressed miRNAs in CRC

miRNA	Genes
hsa-miR-29c-3p	COL1A1, COL1A2, COL4A1, COL5A2, SPARC
hsa-miR-29b-3p	COL1A1, COL4A1, COL5A2, SPARC
hsa-miR-29a-3p	COL4A1, COL5A2, SPARC
hsa-miR-767-5p	COL4A1, COL5A2, SPARC

### SPARC is overexpressed in human CRC specimens

**Figure 3:**
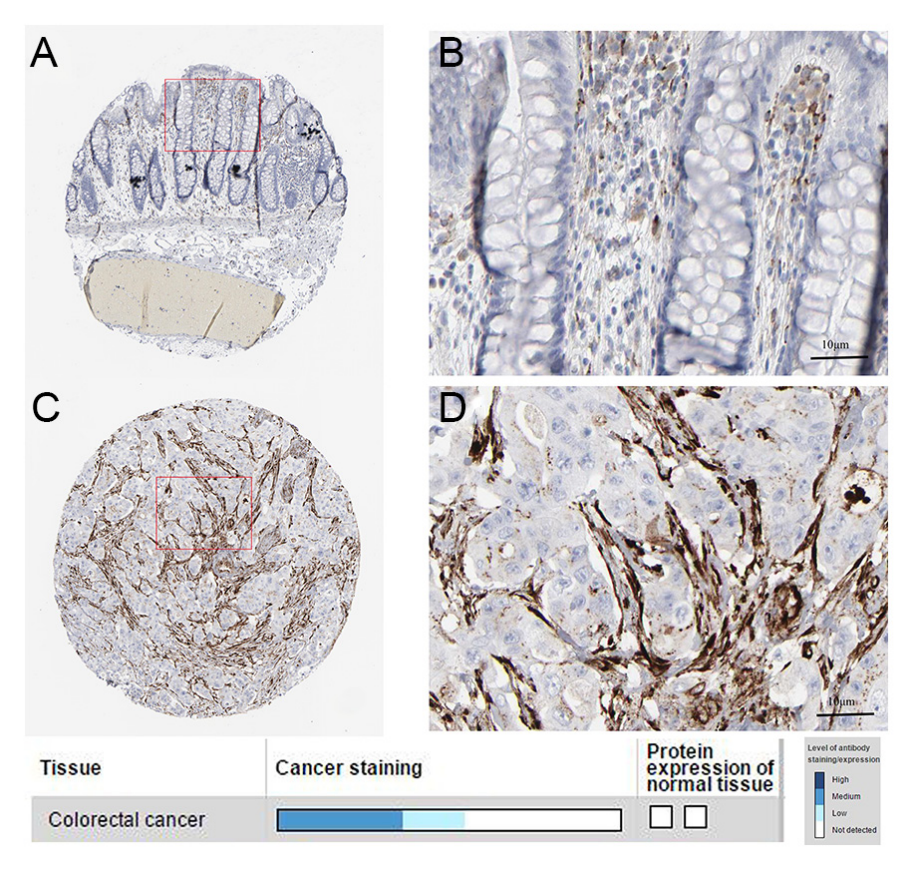
Immunohistochemistry **(A, B)**. normal tissue. **(C, D)** cancer tissue.

To determine the clinical relevance of SPARC expression, we first analyzed the SPARC protein expression in clinical specimens from the human protein atlas (www.proteinatlas.org). We found that SPARC had the strong expression in CRC tissues, and weak expression in normal tissues (Figure 3).

### The expression of proteins and mRNA of these common differential genes in tissues

**Figure 4:**
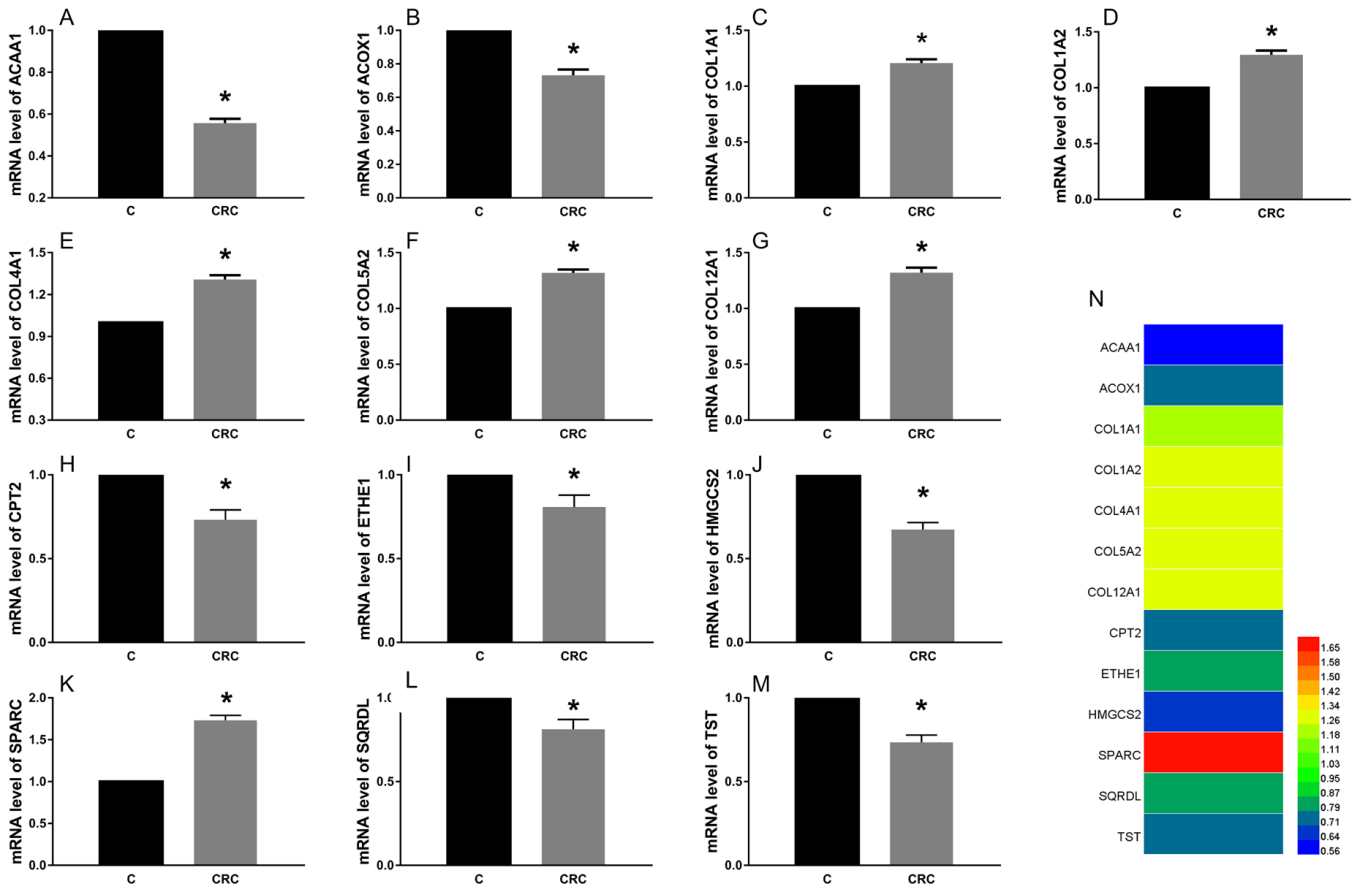
The expression of mRNA of these common differential genes in tissues The expression levels of mRNA COL12A1 **(G)**, COL1A2 **(D)**, COL4A1 **(E)**, SPARC **(K)**, COL5A2 **(F)** and COL1A1 **(C)** was increased, while ACAA1 **(A)**, ACOX1 **(B)**, CPT2 **(H)**, SQRDL **(L)**, HMGCS2 **(J)**, ETHE1 **(I)** and TST **(M)** were decreased. ^*^P < 0.05.

**Figure 5:**
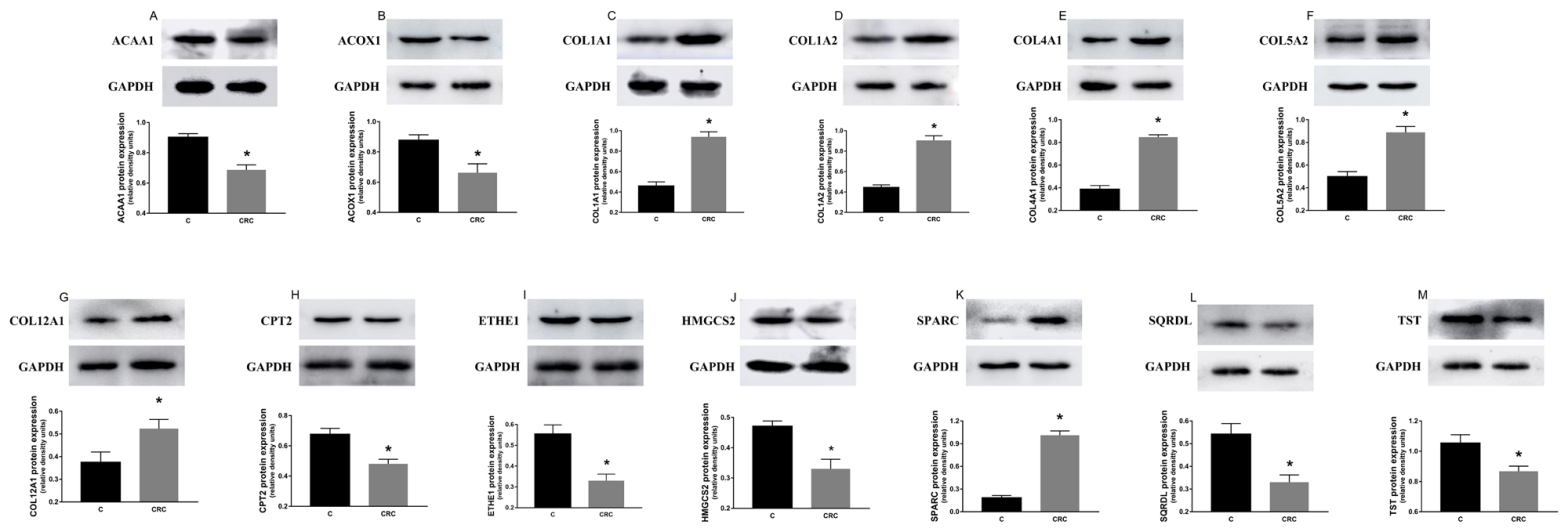
The expression of proteins of these common differential genes in tissues The expression levels of protein COL12A1 **(G)**, COL1A2 **(D)**, COL4A1 **(E)**, SPARC **(K)**, COL5A2 **(F)** and COL1A1 **(C)** was increased, while ACAA1 **(A)**, ACOX1 **(B)**, CPT2 **(H)**, SQRDL **(L)**, HMGCS2 **(J)**, ETHE1 **(I)** and TST **(M)** were decreased. ^*^P < 0.05.

We found that the expression levels of protein and mRNA of these genes were consistent with the results of our bioinformatics analysis (Figure 4, Figure 5). The expression levels of protein and mRNA COL12A1, COL1A2, COL4A1, SPARC, COL5A2 and COL1A1 was increased, while ACAA1, ACOX1, CPT2, SQRDL, HMGCS2, ETHE1 and TST were decreased.

### hsa-miR-29c-3p was down-regulated in colorectal cancer tissues and cell lines

**Figure 6:**
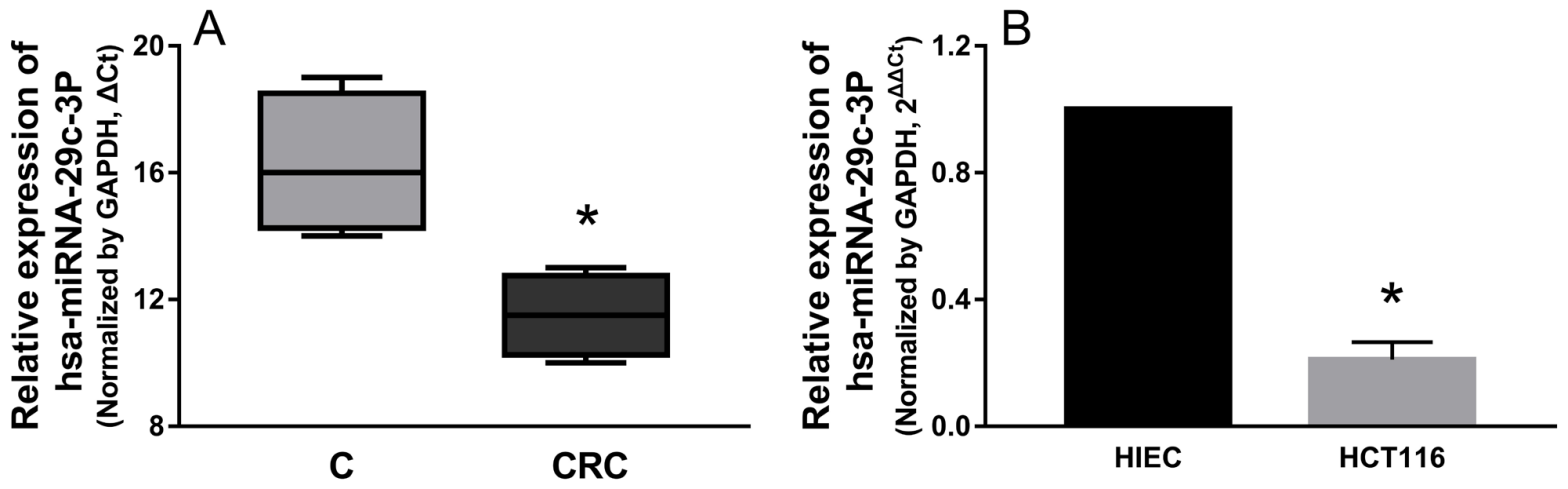
hsa-miR-29c-3p was down-regulated in CRC tissues and cell lines **(A)** The relative expression of hsa-miR-29c-3p in CRC tissues was lower compared with their paired corresponding noncancerous tissues. **(B)** The relative expression of hsa-miR-29c-3p was lower in CRC cells (HCT116 cells) than human epithelial cell line (HIEC) was detected by qRT-PCR. ^*^P < 0.05.

We determined the expression patterns of hsa-miR-29c-3p in CRC tissues, and hsa-miR-29c-3p expression was significantly lower in CRC tissues than in their paired corresponding non-cancerous tissues (Figure 6A), Then, we examined the expression level of hsa-miR-29c-3p in colorectal cancer cell lines (HCT116) and the human epithelial cell line (HIEC), hsa-miR-29c-3p expression was lower in HCT116 cells than the HIEC cells (Figure 6B).

### The effect of hsa-miR-29c-3p on these up-regulated genes

**Figure 7:**
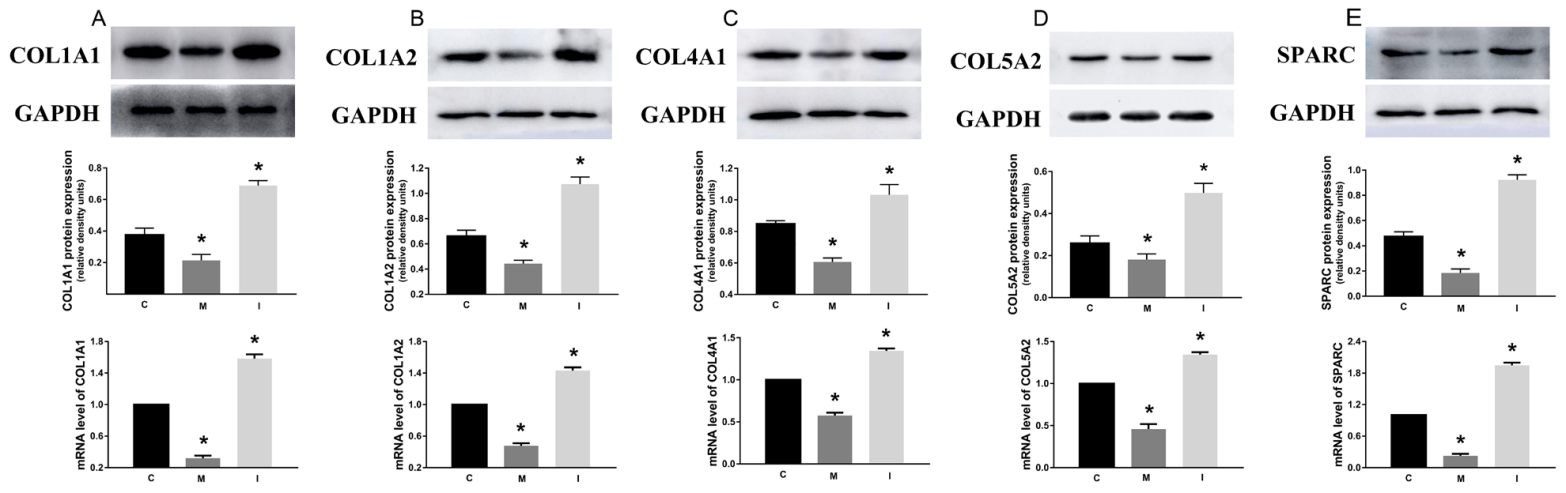
The effect of hsa-miR-29c-3p on these up-regulated genes C group is the control group I group is the hsa-miR-29c-3p inhibitor group. M. The hsa-miR-29c-3p mimic group. the mRNA of COL1A1 **(A)**, COL1A2 **(B)**, COL4A1 **(C)**, COL5A2 **(D)**, SPARC **(E)** was lowest in group I, followed by the group C, the highest was the group M. ^*^P < 0.05.

After HCT116 cells were transfected with hsa-miR-29c-3p mimic, hsa-miR-29c-3p inhibitor, or hsa-miR-29c-3p negative control for 48 h, respectively. qRT-PCR and western blot was used to determine the mRNA and protein level of these five genes. We can see that the mRNA of these genes was lowest in group M, followed by the group C, the highest was the group I. And we found that the greatest degree of change is SPARC, whether inhibited or promoted hsa-miR-29c-3p (Figure 7).

### SPARC was inversed with the expression of hsa-miR-29c-3p in colorectal cancer, as a direct target

**Figure 8:**
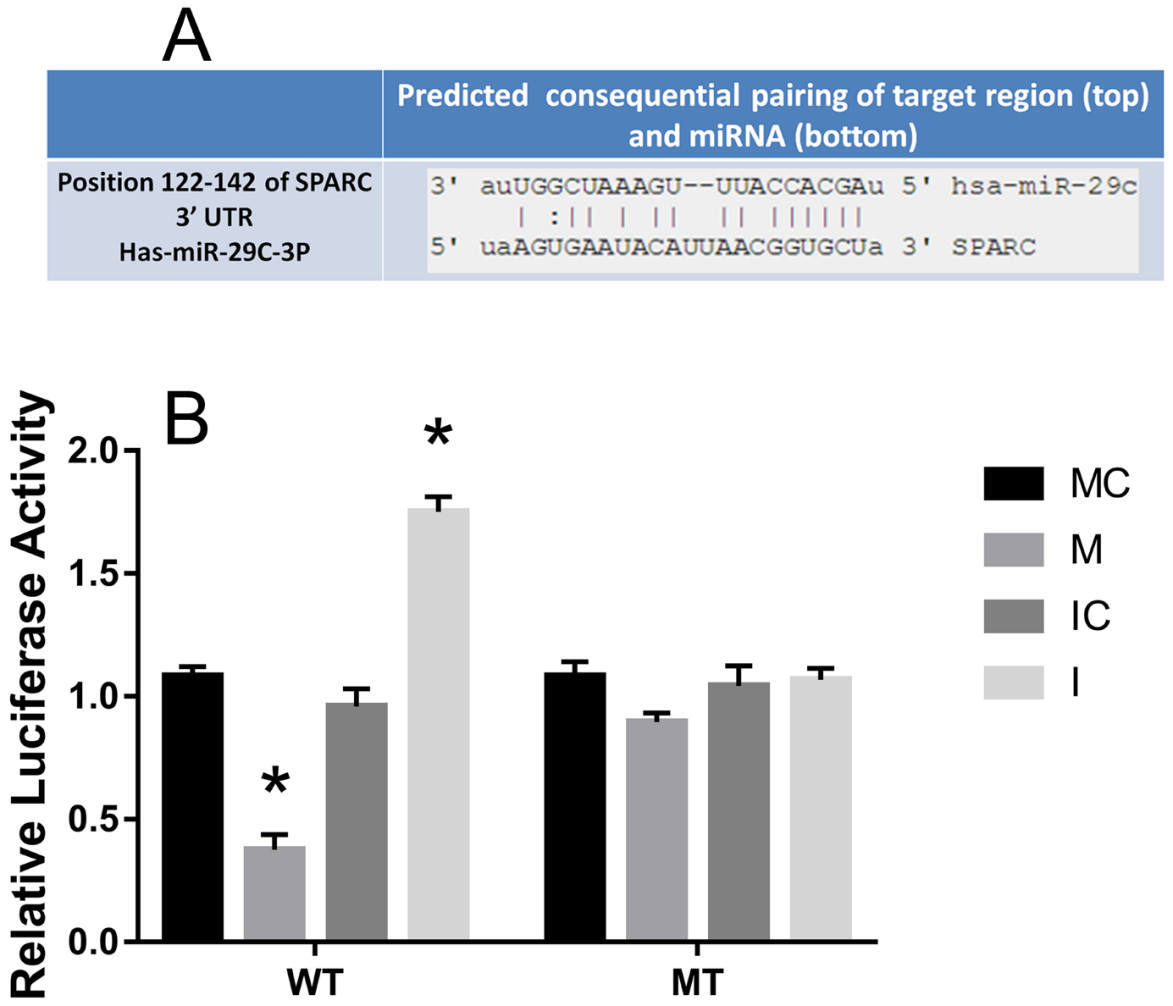
SPARC was inversed with the expression of hsa-miR-29c-3p in CRC, as a direct target **(A)** Predicted SRCIN1 3′-UTR binding site for hsa-miR-29c-3p. **(B)** The relative luciferase activity of the reporter gene in transfected CRC cells. MC group is the 576 mimic control group.M. the hsa-miR-29c-3p mimic group. IC group is the inhibitor control group. I group is the hsa-miR-29c-3p inhibitor group. ^*^P < 0.05.

The predicted interaction between hsa-miR-29c-3p and the target sites in the SPARC 3’UTRwas illustrated in Figure 8A. The SPARC mRNA and protein expression levels were significantly up-regulated in CRC compared to the adjacent normal tissues (Figure 4, Figure 5). Additionally, in CRC cells, hsa-miR-29c-3p mimic significantly reduced the expression of SPARC mRNA and protein, and hsa-miR-29c-3p inhibitor had the opposite effect (Figure 7). To further investigate whether hsa-miR-29c-3p directly and negatively regulates SPARC expression, we constructed luciferase reporter plasmid that contained wild-type (WT) and mutant (MT) hsa-miR-29c-3p target sequences of the SPARC-3’UTR. After co-transfection experiments in CRC cells, we observed a marked reduction in luciferase activity in cells transfected with hsa-miR-29c-3p mimics compared with miR-control-transfected cells (P<0.05). In contrast, no change of luciferase was observed in cells transfected with the mutant 3’-UTR constructs (Figure 8B).

### SPARC knock-down reduce the effect of hsa-miR-29c-3p up-expression in colorectal cancer cell

**Figure 9:**
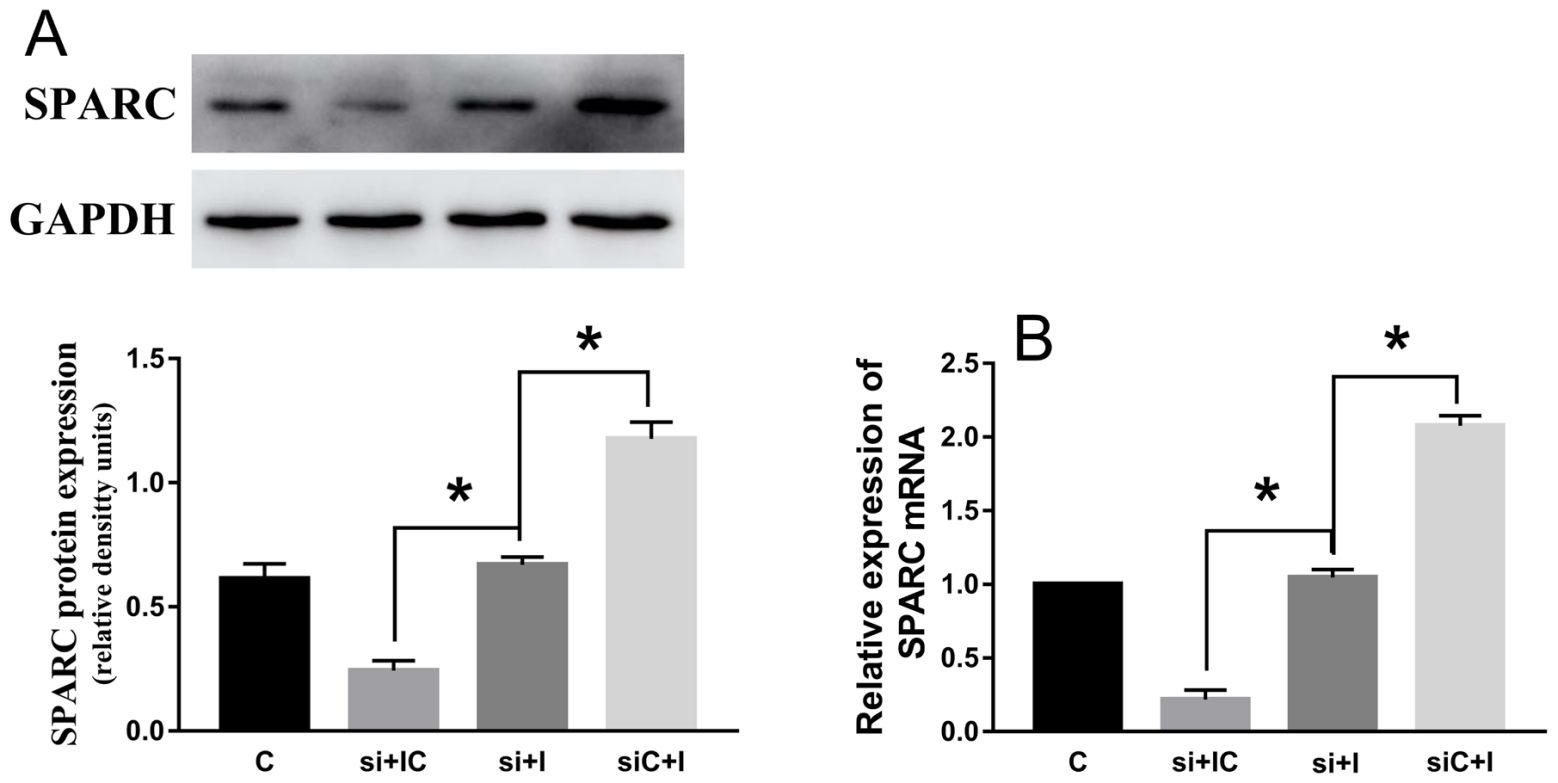
SPARC knock-down reduce the effect of hsa-miR-29c-3p up-expression in colorectal cancer cell **(A)** The protein levels of SPARC in transfected cells were detected by Western blot. **(B)** The relative expression of SPARC mRNA in transfected CRC cell was detected by qRT-PCR. The hsa-miR-29c-3p inhibitor didn’t significantly promoted the expression of SPARC mRNA and protein in co-transfected HCT116 cells. C group is the control group. si + IC group is the si-SPARC + inhibitor control group. si + I group is the si-SPARC + inhibitor group. siC + I group is the si-SPARC control + inhibitor group. ^*^P < 0.05.

After si-SPARC and hsa-miR-29c-3p inhibitor were successfully transfected into HCT116 cells, hsa-miR-29c-3p inhibitor didn’t significantly promoted the expression of SPARC mRNA and protein in co-transfected HCT116 cells (Figure 9A and 9B).

### The effect of hsa-miR-29c-3p and SPARC on cell migration and proliferation

**Figure 10:**
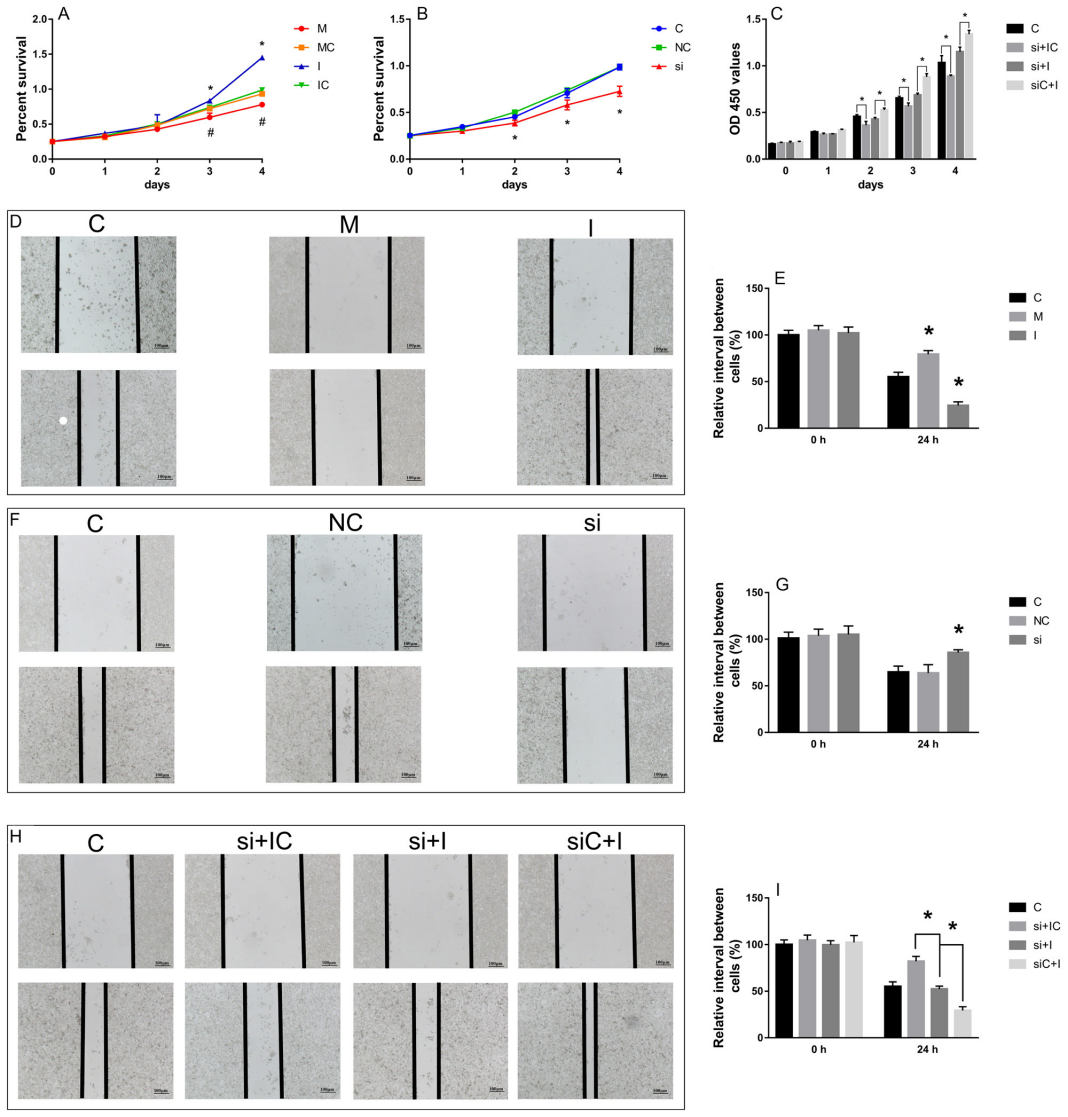
The effect of hsa-miR-29c-3p and SPARC on cell migration and proliferation **(A-C)** the CCK-8 assay. **(D-I)** the wound healing assay. ^*^P < 0.05 (A) C group is the control group. I group is the hsa-miR-29c-3p inhibitor group. M. the hsa-miR-29c-3p mimic group. (B) C group is the control group. NC group is the negative control group. si group is the si-SPARC group. (C) C group is the control group. si + IC group is the si-SPARC + inhibitor control group. si + I group is the si-SPARC + inhibitor group. siC + I group is the si-SPARC control + inhibitor group.

We examined the effect of hsa-miR-29c-3p and SPARC on HCT116 cell migration and proliferation by wound healing assay and CCK-8 assay, respectively. The wound healing assay showed that compared with the control, the hsa-miR-29c-3p mimic and si-SPARC inhibited CRC cell migration capability, while the hsa-miR-29c-3p inhibitor enhanced it (Figure 10D, 10F, 10E, 10G). The CCK-8 assay showed that hsa-miR-29c-3p mimic and si-SPARC inhibited proliferation, in contrast, hsa-miR-29c-3p inhibitor had the opposite effect on cell proliferation (Figure 10A, 10B). Furthermore, we demonstrate that hsa-miR-29c-3p regulates the expression of SPARC mRNA to effect CRC cell proliferation and migration. We first silenced SPARC in CRC cells using siRNA, and then transfected the cells with hsa-miR-29c-3p inhibitor. Then, we examined migration and proliferation ability of the cells. The wound healing and CCK-8 assays showed si-SPARC significantly inhibited colorectal cancer cell proliferation and migration, hsa-miR-29c-3p inhibitor had the opposite effect, however, in the cells co-transfected with hsa-miR-29c-3p inhibitor and the si-SPARC, hsa-miR-29c-3p inhibitor didn’t have significantly effect in cell proliferation and migration (Figure 10C, 10H, 10I). These findings demonstrate that hsa-miR-29c-3p regulates the expression of SPARC mRNA to effect CRC cell proliferation and migration.

## DISCUSSION

In this study, we found that hsa-miR-29c-3p inhibited the biological function of colorectal cancer cells by targeting SPARC. We chose two profiles (GSE4107 and GSE32323) in GEO database, and screened 183 differentially expressed genes between CRC and healthy samples with GEO2R. GO and KEGG pathway enrichment analysis were used in these 183 DEGs. Integration of PPI network and module analysis were in progress to screen 13 hub genes, consisting of 6 up-regulated genes and 7 down-regulated genes. We predicted mRNA targets with miRWalk2.0 and found that the hsa-miR-29c-3p was very important to regulate 5 hub genes. After cell transfection, we found that the change of SPARC was the most obvious, then speculated that it was the main target gene. We also did experiments on CRC cell proliferation and migration after cell transfection. Therefore, we hypothesized that hsa-miR-29c-3p could affect the proliferation and migration of CRC cells through SPARC as a direct target.

Globally, CRC is one of the third most common causes of death by neoplasm and the most common malignant tumor diagnosis in western [28]. CRC, in most cases, is curable when it is detected in its early stages. Functional and KEGG pathway enrichment analysis revealed that genes in this module were mainly associated with protein digestion and absorption, ECM-receptor interaction, fatty acid degradation, fatty acid metabolism, focal adhesion, PPAR signaling pathway, PI3K-Akt signaling pathway, platelet activation and biosynthesis of unsaturated fatty acids, which were closely related to cancer. These 13 hub genes were validated by WB and PCR *in vivo* and *in vitro* experiments and the results are consistent with our software analysis. It was showed that COL4A1 were overexpressed in papillary thyroid carcinoma [29]. The expression of ACOX1 is different according to the location of metastasis, and the lowest expression is in the liver metastasis of breast cancer [30]. HMGCS2 was underexpressed were subsequently verified, which can be served as biomarkers for Esophageal squamous cell carcinoma [31].

It is important that a crucial regulatory role of miRNAs in gene regulatory networks was to maintain balance control. Their regulatory role were essential, and the effects of various biological phenomena in cancer including activation of signaling pathways, proliferation, apoptosis, differentiation, angiogenesis, metastasis and invasion [32]. Alterations of miRNAs were also involved in many diseases such as cardiovascular diseases, neurological disorders, various types of cancer, and autoimmune diseases [33, 34]. It is worth mentioning that previous studies suggested that hsa-miR-29c-3p plays an important role in several pathological processes including Alzheimer’s disease [35], diabetes mellitus [36], abdominal aortic aneurysm [37], and metabolic processes [38]. Recent reports suggested that miRNAs played a key role in the development and progression of various human cancers [39]. And miR-29c is a tumor suppressor gene in miRNAs and its expression is down-regulated has been reported in various types of human cancers. In cancer, hsa-miR-29c-3p was down-regulated in head and neck squamous cell carcinoma [40], and meningioma [41]. Identified miR-29c targets include CDK6 in RCC2 in gastric carcinoma [42], TIAM1 in nasopharyngeal carcinomac [43] and mantle cell lymphoma [44]. Although the calculation analysis showed that miR-29c was related to the early colon cancer recurrence [45], miR-29c can inhibit the metastasis of colon cancer [19] by PTP4A and GNA13. More details about colon cancer miR-29c remain to be revealed, especially in identifying new target genes, so that we can better understand this important role of miRNA in complex regulatory networks. So we use the software for miRNA analysis, found that hsa-miR-29c-3p was very important, and our results show that it had greatest impact on SPARC. *in vivo*, we found that hsa-miR-29c-3p expression was significantly lower in CRC tissues than in their paired corresponding noncancerous tissues. *in vitro*, it was shown that hsa-miR-29c-3p expression was lower in HCT116 cells than the HIEC cells, and hsa-miR-29c-3p inhibited the function of CRC cells. These results were the same as listed above and implied that hsa-miR-29c-3p acted as a tumor suppressor gene in the pathogenesis of CRC.

SPARC is a highly conserved extracellular matrix protein, although it is often a secreted glycoprotein, it is expressed both on cell surface and the intracellular compartment [21]. It can control a variety of biological activities including cell motility, angiogenesis, and adhesion, as well as extracellular matrix remodeling [46, 47]. Tumor associated stroma, including fibroblasts, immune cells, blood vessels and ECM, is crucial for supporting and promoting cancer. It was reported that SPARC was predominantly found in the tumour-associated stroma, specifically in the cytoplasm and ECM of stromal fibroblasts [48, 49]. And SPARC plays an important role in the occur of cancer and tumor progression. SPARC suppresses angiogenesis of gastric cancer by down-regulating the expression of VEGF and MMP-7 [50]. In neuroblastoma cells, overexpression of SPARC can inhibit endothelial cell formation and cell proliferation, including induction of programmed cell death and the inhibition of angiogenic factors expression, such as FGF VEGF, PDGF, and MMP-9 in endothelial cells [49]. SPARC was up-regulated in Barrett’s-associated adenocarcinoma and promoted tumor development [26]. Cancer cells have the characteristics of invasion and migration. The migration and invasion of cells through the basement membrane, distant transmission via the lymphatic or vascular system is an inherent feature of malignant disease. In fact, the interaction between tumor and surrounding tumor stroma is the essence of tumor growth, differentiation, development and metastasis and determines the invasiveness of the tumor [51]. The expression of interstitial SPARC is often characterized by a wide range of tumor necrosis, acidity, hypoxia and oxidative stress, which are the main features of an aggressive tumor [52]. SPARC is beneficial to the growth of tumor and the interaction between tumor and stroma, which is contributing to malignant invasion of tumor [21]. In our study, we found that SPARC was up-regulated and promote cancer cell proliferation and migration via PCR, western-blot, immunohistochemical analysis, CCK-8 assay and wound healing assay. So we also determined SPARC as a oncogene. Recently, SPARC was found to be regulated by several miRNAs. SPARC was targeted by miR-29b, and the down-regulation of mRNA can contribute to the invasion ability [53-55]. miR-203 controls head and neck squamous cell carcinoma metastasis by targeting a network of prometastatic proteins, including LASP1, SPARC, and NUAK1 [56]. It was suggested miR-148b and -152 that are modifying methylation status of tumor suppressor genes such as BNIP3 and SPARC can be applied in killing the pancreatic cancer cells and decreasing the tumorigenicity of these cells [57]. Our data shown that SPARC expression was significantly higher in CRC tissues and cells, and there was a significant inverse correlation with hsa-miR-29c-3p. According to MiRWalk2.0 date, we predicted SPARC as a target of hsa-miR-29c-3p. *in vitro*, we found that hsa-miR-29c-3p inhibited the expression of SPARC, as a direct target, in the CRC cells via PCR, western blot, cell transfection and dual luciferase activity assay. Moreover, we showed that SPARC significantly promoted CRC cell proliferation and migration, and reverse the effect of hsa-miR-29c-3p. These data confirmed that SPARC functioned as a oncogene and hsa-miR-29c-3p as a tumor suppressor gene, and suggested that hsa-miR-29c-3p might influence the downstream pathways by targeting SPARC to affect CRC cell functions.

In summary, we firstly confirmed that the expression of hsa-miR-29c-3p was lower in CRC, inhibited cell migration and proliferation, by targeting SPARC, which revealed a critical role and appeared to be a promising therapeutic target.

## MATERIALS AND METHODS

### Microarray data

Two gene expression profiles (GSE4107 and GSE32323), species is human. The array data of GSE4107 included 12 CRC tissue samples and 10 healthy samples which were analyzed using GeneChip U133-Plus 2.0 Array. GSE32323 had 17 pairs of cancer and non-cancerous tissues from CRC patients were measured by Affymetrix Human Genome U133 Plus 2.0 Array.

### Data processing

The GEO database archives a large number of high throughput functional genomic studies that contain data that are processed and normalized using various methods. GEO2R (http://www.ncbi.nlm.nih.gov/geo/geo2r/) was applied to screen differentially expressed genes between CRC and healthy samples. GEO2R performs comparisons on original submitter-supplied processed data tables using the GEO query and limma R packages from the bioconductor project. The adjusted P values (adj. P) were applied to correct for the occurrence of false positive results using Benjamini and Hochberg false discovery rate method by default. The adj. P < 0.05 and |logFC| > 1 were set as the cut-off criterion.

### Functional and pathway enrichment analysis

The Database for Annotation, Visualization and Integrated Discovery (DAVID, https://david.ncifcrf.gov/home.jsp) provides a comprehensive set of functional annotation tools for investigators to understand biological meaning behind large list of genes. GO and KEGG pathway enrichment analysis were performed for identified DEGs using DAVID database. P<0.05 was set as the cut-off criterion.

### Integration of protein-protein interaction (PPI) network and module analysis

The functional interactions between proteins can provide context in molecular mechanism of cellular processing. In present study, PPI network of DEGs was constructed using the Search Tool for the Retrieval of Interacting Genes (STRING, http://string-db.org) database and subsequently was visualized using Cytoscape (http://www.cytoscape.org/index.html). And confidence score > 0.4 was set as the cut-off criterion. Then, the Molecular Complex Detection (MCODE) was performed to screen modules of PPI network with degree cutoff = 2, node score cutoff = 0.2, K-Core = 2 and Depth from Seed = 100.

### Prediction of mRNA targets

miRWalk2.0 (http://zmf.umm.uni-heidelberg.de/apps/zmf/mirwalk2/miRretsys-self.html) is freely accessible, comprehensive archive, supplying the biggest available collection of predicted and experimentally verified mRNA-target interactions with various novel and unique features to assist the mRNA research community, which is an integrated resource produced by established mRNA target prediction programs. The genes predicted by miRWalk, RNA22, miRanda and Targetscan programs were identified as the targets of mRNAs.

### Analysis of SPARC protein expression in human CRC

SPARC protein expression in CRC tissues and normal tissues was determined from 15

### Cell culture and clinical tissues

Human colon cancer cell lines HCT116 an immortalized human epithelial cell line (HIEC) were cultured in RPMI 1640 (Life Technologies Inc., Cergy Pontoise, France) supplemented with 10% fetal bovine serum (FBS; Gibco, Grand Island, NY, USA) and 1% penicillin/streptomycin (P/S; Sigma, St. Louis, MO, USA). HIEC cells were also supplemented with human insulin (0.1 U/mL). The cells were incubated at 37°C in a humidified atmosphere of 5% CO_2_. Tissue samples were collected from 20 patients (diagnosed at the First Affiliated Hospital of Henan University of Science and Technology, from 2012 to 2017, 10 men and 10 women, aged 65-75) with CRC as a test set. In each case, grossly normal mucosa remote from the tumor was included as a control. All of the patients were given written informed consentand the study was approved by the Ethics Committee of the First Affiliated Hospital of Henan University of Science and Technology.

### Cell transfection

HCT116 cells were plated in 6-well plates (2.5 × 10^5^ cell/well) for 24 h, then transfected with human hsa-miR-29c-3p mimic, hsa-miR-29c-3p inhibitor, hsa-miR-29c-3p negative control (GenePharma, Shanghai, China), small inhibitory RNA (siRNA) against human-SPARC (si-SPARC) and NC-siRNA (RIBOBIO, Guangzhou, China) using Lipo2000 (Applied Biosystems, Life Technologies, USA) (Table 4). 12 h–48 h after transfected with miRNA or siRNA, cells were harvested for the next experiment. The efficiency of transfection was confirmed by hsa-miR-29c-3p and protein expression using qRT-PCR and Western Blot, respectively.

**Table 4:** Primer pairs of different genes

Gene	Primer pairs	
hsa-miR-29c-3p mimics	Sense (5’-3’)	UAGCACCAUUUGAAAUCGGUUA
	Antisense (5’-3’)	ACCGAUUUCAAAUGGUGCUAUU
hsa-mir-29c-3p inhibitor	Sense (5’-3’)	UAACCGAUUUCAAAUGGUGCUA
	Antisense (5’-3’)	
Si-SPARC	Sense (5’-3’)	AUGAGGACAACAACCUUCUTT
	Antisense (5’-3’)	AGAAGGUUGUUGUCCUCAUCC

### Dual luciferase activity assay

To detect the binding specificity, we constructed the wild-type and mutant seed region of SPARC 3’UTR, which contained the putative target site for the mature hsa-miR-29c-3p, and cloned the minto the pGL3-control vector. HCT116 cells were co-transfected with hsa-miR-29c-3p mimic, inhibitor or negative control, pGL3 -SPARC 3’UTR-WT vector, pGL3-SPARC 3’UTR-MT vector and phRL-SV40 control vector (Promega, USA) using Lipo2000 (Applied Biosystems, Life Technologies, USA) in 24-well plates. After transfected for 24 h, the relative luciferase activity was detected by the Dual-Luciferase Reporter Assay System (Promega, USA) and normalized with renilla luciferase activity using SpectraMax i3 (Molecular Devices, USA).

### Wound healing assay

After 6 h transfection with hsa-miR-29c-3p mimic or inhibitor, negative siRNA, or si-SPARC, 2 ×105 cells per well were seeded into 6-well plates and cultured in RPMI 1640 medium containing 10% FBS and 1% P/S at 37°C in a humidified incubator with 5% CO2 to synchronize the cells. A wound for 24 h until 90% confluent, and then the medium was changed to medium containing 0.05% FBS and 1% P/S overnight was then created in the cell monolayer using a 100-μL yellow pipette tip. The wound areas were washed with phosphate-buffered saline and photographed with an inverted light microscope (Olympus IX51, Center Valley, PA, USA) at 0 h and 24 h. The ratio of the remaining wound area relative to the initial wound area was calculated and the wound area was quantified using Image-Pro Plus v. 6.0 software (Media Cybernetics, Bethesda, MD, USA).

### CCK-8 assay

Cell proliferation was detected by the Cell Counting Kit-8 (CCK-8). 24 h after the transfection with miRNA or si-SPARC, HIEC cells (2.0 × 10^3^) were plated into 96-well plates and cultured for 1, 2, 3 or 4 days, in addition, HCT116 cells (5.0×10^3^) were plated into 96-well plates then discarded the old culture media, 10 μl CCK-8 (Dojindo, Japan) in 100 μl culture medium was added to each well and incubated for a further 1 h at 37 °C. The absorbance was measured at a wavelength of 450 nm.

### Real-time RT-PCR

Total RNA (n = 4) was isolated from tissues and cells with Trizol reagent (Applied Biosystems, Invitrogen, USA). The total RNA was reverse transcribed (Prime Script RT Master Mix; Takara Bio Inc., Shiga, Japan) according to the manufacturer’s protocol. Quantitative real-time RT-PCR was performed using an ABI Prism 7 000 (Life Technologies). Reactions were performed in 20 μL of reaction mixture containing 10 μL PCR master mix (SYBR Premix Ex Taq II; Takara Bio Inc.), 0.4 μL primer pairs (Table 5), and 2 μL cDNA samples. After normalization with reference to expression of GAPDH, the relative expression levels of hsa-miR-29c-3p and SPARC were calculated by the ΔCt or 2^−ΔΔCt^ method.

**Table 5:** Primer pairs of different genes

Gene		Primer pairs
ACAA1	FORWARD	CATAGCAGGTGGCATCAGAA
	REVERSE	AGGGCAAAGGTATCCTGCTT
ACOX1	FORWARD	GCCTCTGGATCTTCACTTGG
	REVERSE	GTCTGGGCATAAGTGCCAAT
COL1A1	FORWARD	CCTGGATGCCATCAAAGTCT
	REVERSE	AATCCATCGGTCATGCTCTC
COL1A2	FORWARD	ACCTGGTCAAACTGGTCCTG
	REVERSE	CCTGTGGTCCAACAACTCCT
COL4A1	FORWARD	CTGGTCCAAGAGGATTTCCA
	REVERSE	TCATTGCCTTGCACGTAGAG
COL5A2	FORWARD	GAGCTGGGAAACGTGGATTA
	REVERSE	CAGGAAGACCCTGAAGACCA
COL12A1	FORWARD	AATTGCCTCCACACCTTCAC
	REVERSE	TCACCAAGCTGCTCATCAAC
CPT2	FORWARD	GAGCTCAGGCAGAAGCTGAT
	REVERSE	GTGGGACAAGTGGACAAGGT
ETHE1	FORWARD	CTGTGTCACCTTCGTCCTGA
	REVERSE	ATGGACCGAGTGGTACAAGG
HMGCS2	FORWARD	GGACCAAACTGACCTGGAGA
	REVERSE	GTCAGGCACAGGGAGTTGAT
SPARC	FORWARD	GCTGTGTTGGAAACGGAGTTG
	REVERSE	CTTGCCATGTGGGTTCTGACT
SQRDL	FORWARD	AGACCAGTCCTGTGGCTGAT
	REVERSE	GCGGTCTTTGACGTAGGAAG
TST	FORWARD	GATCTCTCGCAGCCTCTCAT
	REVERSE	GACCAGGAGCCATCGTACAC
GAPDH	FORWARD	GCACCGTCAAGGCTGAGAAC
	REVERSE	TGGTGAAGACGCCAGTGGA

### Western blot

Briefly, total proteins (n = 4) were extracted with RIPA buffer (contain 1% PMSF) and quantification by BCA kit (P0010, Beyotime, China), an equal quantity of protein from each sample (20–60 μg) was separated by 10% sodiumdodecyl sulfate polyacrylamide gel electrophoresis, and transferred onto PVDF and NC membranes. Membranes were performed blockage whit 5% skim milk for 3 h and then incubation with primary antibodies for ACAA1 (ab110289, Abcam, 1:1000) antibody, ACOX1 (ab184032, Abcam, 1:1000) antibody, COL1A1 (PB0980, Boster, 1:500) antibody, COL1A2 (ab208638, Abcam, 1:500) antibody, COL4A1 (PB0126, Boster, 1:500) antibody, COL5A2 (A03869, Boster, 1:1000) antibody, COL12A1 (ab121304, Abcam, 1:1000) antibody, CPT2 (ab181114, Abcam, 1:4000) antibody, ETHE1 (ab174302, Abcam, 1:4000) antibody, HMGCS2 (ab137043, Abcam, 1:4000) antibody, SPARC (ab207743, Abcam, 1:1000) antibody, SQRDL (ab71978, Abcam, 1:500) antibody, TST (ab166625, Abcam, 1:4000) antibody and Glyceraldehyde-3-phosphate dehydrogenase (GAPDH) (ab8245, Abcam, 1:4000) antibody, and subsequent incubation whit horseradish peroxidase (HRP)-coupled goat anti-rabbit (ZDR-5306, Beijing ZSBio, 1:5000) or HRP-coupled goat anti-mouse (W420B, Promega, 1:5000) secondary antibody, respectively. The protein bands were detected using an enhanced chemiluminescence detection system ECL (Biological Industries, Kibbutz Beit Haemek, Israel). Gray value analysis of the protein bands was performed using ImageJ software (National Institutes of Health, Bethesda, MD, USA). GAPDH was used as the loading control.

### Statistical analysis

All statistical parameters were calculated using GraphPad Prism 7.0 software. Values are expressed as the mean±S.D. Comparisons of two groups were performed using Student’s *t*-tests; more than 2 independent groups were compared using one-way analysis of variance. P < 0.05 was considered statistically significant.
